# Functional Selectivity Does Not Predict Antinociceptive/Locomotor Impairing Potencies of NOP Receptor Agonists

**DOI:** 10.3389/fnins.2021.657153

**Published:** 2021-03-30

**Authors:** Joaquim Azevedo Neto, Chiara Ruzza, Chiara Sturaro, Davide Malfacini, Salvatore Pacifico, Nurulain T. Zaveri, Girolamo Calò

**Affiliations:** ^1^Section of Pharmacology, Department of Neuroscience and Rehabilitation, University of Ferrara, Ferrara, Italy; ^2^Technopole of Ferrara, LTTA Laboratory for Advanced Therapies, Ferrara, Italy; ^3^Department of Chemical and Pharmaceutical Sciences, University of Ferrara, Ferrara, Italy; ^4^Astraea Therapeutics, LLC., Mountain View, CA, United States; ^5^Department of Pharmaceutical and Pharmacological Sciences, University of Padua, Padua, Italy

**Keywords:** NOP receptor, nociceptin/orphanin FQ, functional selectivity, mice, β arrestin 2 knockout mice, MCOPPB, Ro 65-6570, AT-403

## Abstract

Nociceptin/orphanin FQ controls several functions, including pain transmission, via stimulation of the N/OFQ peptide (NOP) receptor. Here we tested the hypothesis that NOP biased agonism may be instrumental for identifying innovative analgesics. *In vitro* experiments were performed with the dynamic mass redistribution label free assay and the NOP non-peptide agonists Ro 65-6570, AT-403 and MCOPPB. *In vivo* studies were performed in wild type and β-arrestin 2 knockout mice using the formalin, rotarod and locomotor activity tests. *In vitro* all compounds mimicked the effects of N/OFQ behaving as potent NOP full agonists. *In vivo* Ro 65-6570 demonstrated a slightly higher therapeutic index (antinociceptive vs. motor impairment effects) in knockout mice. However, all NOP agonists displayed very similar therapeutic index in normal mice despite significant differences in G protein biased agonism. In conclusion the different ability of inducing G protein vs. β-arrestin 2 recruitment of a NOP agonist cannot be applied to predict its antinociceptive vs. motor impairment properties.

## Introduction

The peptide nociceptin/orphanin FQ (N/OFQ) is the endogenous ligand of the N/OFQ peptide (NOP) receptor (Meunier et al., 1995; Reinscheid et al., 1995). The N/OFQ-NOP receptor system regulates several biological functions, including pain transmission, locomotor activity, memory, emotional states, food intake, drug abuse, micturition, cough reflexes, cardiovascular, respiratory, and immune functions (Lambert, 2008; Toll et al., 2016). The NOP receptor represents an innovative pharmacological target for the treatment of several conditions, including pain (Schröder et al., 2014; Calo and Lambert, 2018), depression (Gavioli and Calo’, 2013), drug addiction (Ciccocioppo et al., 2019), Parkinson’s disease (Mercatelli et al., 2020), and incontinence due to overactive bladder (Angelico et al., 2019). However, the pleiotropic effects exerted by N/OFQ and NOP ligands may limit their drug development. For instance, the sedative effects associated with high doses of NOP agonists hampered their development as antitussives

(Woodcock et al., 2010) or analgesics (Byford et al., 2007). We hypothesized that one way to trigger some of the NOP mediated effects instead of others, might be the so-called functional selectivity of NOP agonists.

Functional selectivity, or biased agonism, is the ability of a ligand to stabilize different active conformations of the same receptor and selectively activate different downstream signaling pathways (i.e., promoting the interaction of the receptor with G protein over arrestin recruitment or vice versa). Biased agonists have been proposed as “smarter drugs,” with the potential of specifically targeting therapeutic signaling pathways while avoiding others that could lead to side effects (Kenakin, 2019). In recent years biased ligands have been identified and characterized for several different G protein coupled receptors (Tan et al., 2018); however, despite the high potential of the biased agonism strategy in drug discovery, we still need to substantially increase our knowledge regarding translation of *in vitro* bias to *in vivo* drug effects (Michel and Charlton, 2018; Kenakin, 2019). With the NOP receptor, *in vitro* studies have been performed to investigate the ability of NOP ligands to differently recruit G protein and β-arrestin (Chang et al., 2015; Malfacini et al., 2015; Ferrari et al., 2016, 2017; Pacifico et al., 2020). Additionally, there is *in vitro* and *in vivo* evidence that NOP agonists are able to differently activate G protein-dependent events and receptor phosphorylation/and internalization (Mann et al., 2019). As far as *in vivo* impact of NOP functional selectivity is concerned, the only information available suggests that NOP ligands producing similar effects on NOP/G protein interaction but showing different effects on β-arrestin 2 recruitment, elicited different actions on anxiety and mood (Asth et al., 2016).

**FIGURE 1:**
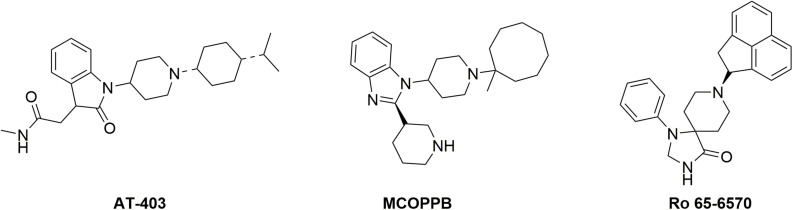
Chemical structure of the NOP agonists used in this study.

The aim of the present study was to investigate the *in vivo* impact of NOP receptor functional selectivity; in particular we tested the hypothesis that NOP biased agonism may be instrumental for identifying effective analgesics devoid of the locomotor impairing effects associated with NOP activation. To this aim we integrated the results of *in vitro*, and genetic and pharmacological *in vivo* studies: (i) NOP selective agonists (Ro 65-6570, AT-403, and MCOPPB) characterized by different degree of biased agonism toward G protein (Ferrari et al., 2017) were studied and compared in the dynamic mass redistribution (DMR) test, a label-free assay recently validated for the NOP receptor in our laboratories (Malfacini et al., 2018), (ii) the antinociceptive and motor effects of Ro 65-6570 were evaluated in wild type and in mice knockout for the β-arrestin2 gene [βarr2(−/−)], and iii) dose response curves for antinociceptive and motor effects were performed in normal mice using Ro 65-6570, AT-403, and MCOPPB in order to estimate their therapeutic index. The formalin assay was used as analgesiometric test while locomotor activity and rotarod assays were used to determine locomotor and coordination impairment. The chemical structure of Ro 65-6570, AT-403, and MCOPPB is shown in Figure 1. These NOP agonists have been selected based on their different degree of biased agonism; in fact, a previous study performed in our laboratories demonstrated that AT-403 behaves as an unbiased NOP agonist while Ro 65-6570 and MCOPPB are biased toward G protein with bias factors of 1.64 and 0.97, respectively (Ferrari et al., 2017).

## Materials and Methods

### Drugs

The following drugs were used to perform this study: the NOP agonists N/OFQ and Ro 65-6570 (synthesized and purified in house at the Department of Chemistry and Pharmaceutical Sciences, University of Ferrara, Italy), AT-403 (synthesized at Astraea Therapeutics, Mountain view, CA), MCOPPB trihydrochloride hydrate (Sigma Aldrich, St. Louis, MO, United States), and pertussis toxin (PTX, Tocris Bioscence, Bristol, United Kingdom). N/OFQ was solubilized in 0.9% NaCl water solution. PTX was solubilized in water, stock solution 100 μg/ml. Other compounds were solubilized in water containing 1% DMSO and 0.3% cyclodextrin. NOP ligands were injected intraperitoneally (i.p.), in a volume of 10 ml/kg, 30 min before the test.

### Dynamic Mass Redistribution Assay

DMR experiments were conducted as previously described (Malfacini et al., 2018). Chinese Hamster Ovary (CHO) cells stably expressing the human NOP receptor (CHO_*NOP*_) were kindly provided by D.G. Lambert (University of Leicester, United Kingdom). Confluent cells were sub-cultured using trypsin/EDTA and used for experiments. CHO cells were used as control. Cells were cultured in Dulbecco’s Modified Eagle Medium: Nutrient Mixture F-12 (DMEM/F12) supplemented with 10% (v/v) Fetal Calf Serum (FCS), 100 U/ml penicillin, 100 μg/ml streptomycin, 2 mM L-glutamine, 15 mM HEPES. The medium was supplemented with 400 μg/ml G418 to maintain expression. Cells were seeded at a density of 45,000 cells/well in 120 μl into fibronectin-coated Enspire^TM^-LC 96-wells plates and cultured 20 h to form a confluent monolayer. The day of the experiment cells were manually washed twice and maintained with assay buffer [Hank’s Balanced Salt Solution (HBSS) with 20 mM HEPES, 0.01% Bovine Serum Albumin (BSA) fraction V] for 90 min before DMR experiments. DMR was monitored in real time with a temporal resolution of 44 s throughout the assay. Experiments were performed at 37°C, using an EnSight Multimode Plate Reader (PerkinElmer). A 5 min baseline was first established, followed by adding compounds manually in a volume of 40 μl and recording compound-triggered DMR signal for 60 min. PTX (200 ng/ml) was added 20 h before NOP agonists. Maximum picometers (pm) modification (peak) were used to determine agonist response after baseline normalization.

### Animals

All experimental procedures adopted for *in vivo* studies were as humane as possible, complied with the European Communities Council directives (2010/63/E) and Italian regulations (D.Lgs, 26/2014). The study was approved by the Animal Welfare Body of the University of Ferrara and by the Italian Ministry of Health (License N° 302/2017). *In vivo* studies have been reported according to ARRIVE guidelines (Kilkenny et al., 2010). Male CD-1 mice (ENVIGO, Udine, Italy) were used in this study together with mice knockout for the NOP receptor gene [NOP(−/−)] and βarr2(+/+) and βarr2(−/−) mice. Details about the generation of NOP(−/−) mice have been published previously (Nishi et al., 1997; Bertorelli et al., 2002), moreover NOP(+/+) and NOP(−/−) mice have been backcrossed on CD-1 strain in our laboratories. βarr2(−/−) mice were from The Jackson Laboratory [JAX stock #011130; (Bohn et al., 1999)] and C57BL/6J mice were used as controls. All animals were housed and bred in the Animal Facility of the University of Ferrara LARP, in specific pathogen free conditions, and genotyped by PCR as described in details by Holanda et al. (2019) for NOP(−/−) mice and in The Jackson Laboratory protocol 23872, version 1.2. for βarr2(−/−) mice. Mice were housed in 425 × 266 × 155 mm cages (Tecniplast, MN, Italy), under standard conditions (22°C, 55% humidity, 12 h light–dark cycle, lights on 7.00 am) with food (4RF, Mucedola, Italy) and water *ad libitum*. A mouse red house (Tecniplast, VA, Italy) and nesting materials were present in each cage. Mice 8–12 weeks old were used (body weight of 25–30 g for C57BL/6J mice, body weight of 30–35 g for CD-1 mice). Each animal was used only once and killed with CO_2_ overdose at the end of the experiment.

### Rotarod Test

To investigate potential effects on motor coordination we performed a rotarod test using a constant speed device (Ugo Basile, Varese, Italy), as previously described (Rizzi et al., 2016). Mice were trained at 15 rpm for 120 s 1 day before the experiment. Motor performance was calculated as time (sec) spent on the rod. A cut-off time of 120 s was chosen. Ro 65-6570 was injected i.p. 30 min before starting the test. Rotarod experiments have been performed to test the effects of Ro 65-6570 in βarr2(+/+) and βarr2(−/−) C57BL/6J mice.

### Locomotor Activity Test

For locomotor activity experiments the ANY-maze video tracking system was used (Ugo Basile, application version 4.52c Beta), as previously described (Guerrini et al., 2009). Mice were positioned in square plastic cages (40 × 40 cm), one mouse per cage. Four mice were monitored in parallel. Mouse horizontal activity was monitored by a camera while vertical activity was measured by an infrared beam array. The parameters measured were cumulative distance traveled (total distance in m traveled during the test), total time immobile (seconds the animal stays immobile during the test; the animal is considered immobile when 90% of his image remains in the same place for at least 2.5 s), and the number of animal rearings (number of beam breaks due to vertical movements). The test lasts 60 min. NOP agonists were injected i.p. 30 min before starting the test. Locomotor activity test experiments have been performed to test the effects of Ro 65-6570, AT-403, and MCOPPB in CD-1 mice and to test the effects of Ro 65-6570 and AT-403 in NOP(+/+) and NOP(−/−) CD-1 mice.

### Formalin Test

The procedure from Hunskaar and Hole (1987) was established in our laboratories (Rizzi et al., 2006, 2016). Approximately 30 min before testing, mice were individually placed in transparent observations chambers (32 cm high, 24 cm diameter) for adaptation. Then the animal was taken out of the chamber, and 30 μl of a 1.5% formalin solution were injected into the dorsal surface of the right hind paw. Immediately after formalin injection, each mouse was returned to the observation chamber, and time (s) spent by the animal displaying pain-related behaviors was measured with a handheld stopwatch for each 5 min block for 45 min after formalin injection. The nociceptive behaviors consisted of licking, biting and lifting of the injected paw. Time spent by the animal showing all these pain-related behaviors was cumulatively measured and expressed as seconds of nociceptive behavior/min. The cumulative response times during 0–10 min and during 15–45 min were regarded as first and second phase, respectively. NOP agonists were injected i.p. 30 min before formalin injection. Formalin test experiments have been performed to test the effects of Ro 65-6570 in βarr2(+/+) and βarr2(−/−) C57BL/6J mice and to test the effects of AT-403 and MCOPPB in CD-1 mice.

### Data Analysis

The pharmacological terminology adopted in this paper is consistent with IUPHAR recommendations (Neubig et al., 2003). All data were analyzed using Graph Pad Prism 6.0 (La Jolla, CA, United States). *In vitro* studies: concentration-response curves were fitted using the four parameters non-linear regression model.

$$\text{Effect}\;=\;\text{Baseline}\;+\;\left({\text{E}}_{\text{max}}-\text{Baseline}\right)/(\text{1}\;+10^{{{\text{(LogEC}}_{50}-\text{Log[compound])}}_{*}\text{Hillslope}})$$

Data are expressed as mean ± SEM of n experiments performed in duplicate. Agonist potency was expressed as pEC_50_, which is the negative logarithm to base 10 of the agonist molar concentration that produces 50% of the maximal possible effect of that agonist. *In vivo* studies: data are expressed as mean ± S.E.M. of n animals. Data were analyzed using one-way or two-way analysis of variance (ANOVA) followed by Dunnett’s or Bonferroni’s *post hoc* test, as specified in figure legends. Differences were considered statistically significant when *p* < 0.05. For therapeutic index calculation *in vivo* data from formalin (second phase) and locomotor activity (total distance traveled) tests have been expressed as % of control, where control corresponds to vehicle treated animals. Dose-response curves to agonists were fitted to the classical four-parameter logistic non-linear regression model. Bottom and top were constrained to 0 and 100%, respectively. ED_50_ is the dose of the agonist that produces 50% of the maximal effect of that agonist. For each agonist therapeutic index was calculated as the ratio ED_50_ in the locomotor activity test/ED_50_ in the formalin test.

## Results

### Dynamic Mass Redistribution Effects of MCOPPB, Ro 65-6570, and AT-403

**FIGURE 2:**
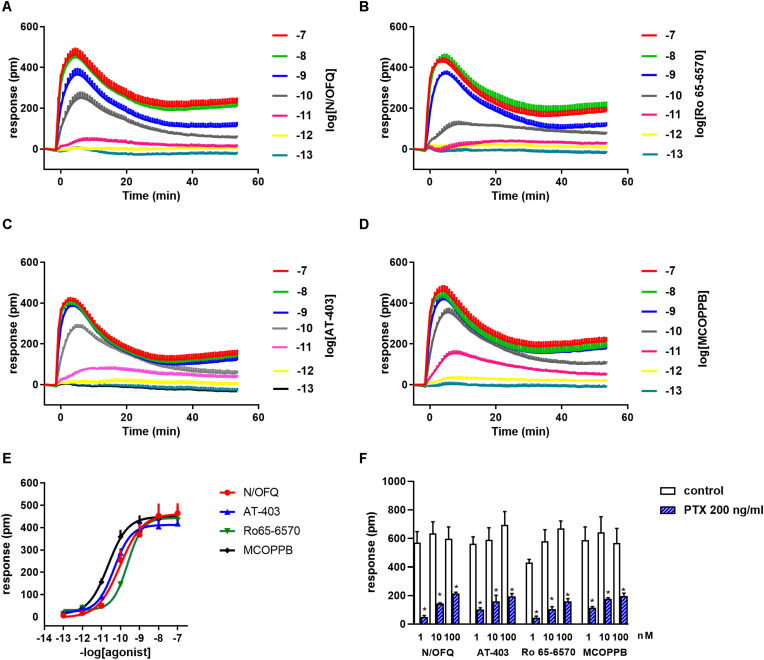
DMR traces in response to N/OFQ **(A)**, Ro 65-6570 **(B)**, AT-403 **(C)**, and MCOPPB **(D)**. Concentration response curves to NOP receptor agonists are displayed in **(E)**. The effects of high concentrations of NOP agonists in the absence and presence of 200 ng/ml PTX are shown in **(F)**. Data are mean ± SEM of 5 experiments performed in duplicate and of 3 experiments performed in duplicate **(F)**. Two-way ANOVA (agonist × PTX) revealed an effect of PTX *F*_(1, 47)_ = 352. *p < 0.05 vs. control, Tukey’s test.

As shown in Figure 2A N/OFQ evoked a concentration dependent DMR response in CHO cells expressing the human recombinant NOP receptor showing high potency (pEC_50_ 9.99, CL_95%_ 9.50–10.48) and maximal effects (464 ± 4 pm). MCOPPB, Ro 65-6570, and AT-403 mimicked the stimulatory effects of N/OFQ showing similar traces and maximal effects (464 ± 31, 434 ± 25, and 418 ± 14 pm, respectively, Figures 2B–D). As far as potency is concerned MCOPPB and AT-403 were slightly more potent than N/OFQ (pEC_50_ 10.55, CL_95%_ 10.03–11.07; pEC_50_ 10.31, CL_95%_ 10.11–10.51, respectively) while Ro 65-6570 was slightly less potent (pEC_50_ 9.63, CL_95%_ 9.54–9.72) (Figure 2E). Importantly 100 nM N/OFQ did not elicit any effect in CHO wild type cells and similar results were obtained for MCOPPB, Ro 65-6570, and AT-403 tested at the same concentration (data not shown). Finally, the DMR response to 1–100 nM N/OFQ in CHO_*NOP*_ cells was nearly abolished by pre-treatment with 200 ng/ml of PTX. Superimposable results were obtained with MCOPPB, Ro 65-6570, and AT-403 (Figure 2F).

### Effects of Ro 65-6570 in βarr2(+/+) and βarr2(−/−) Mice

**FIGURE 3:**
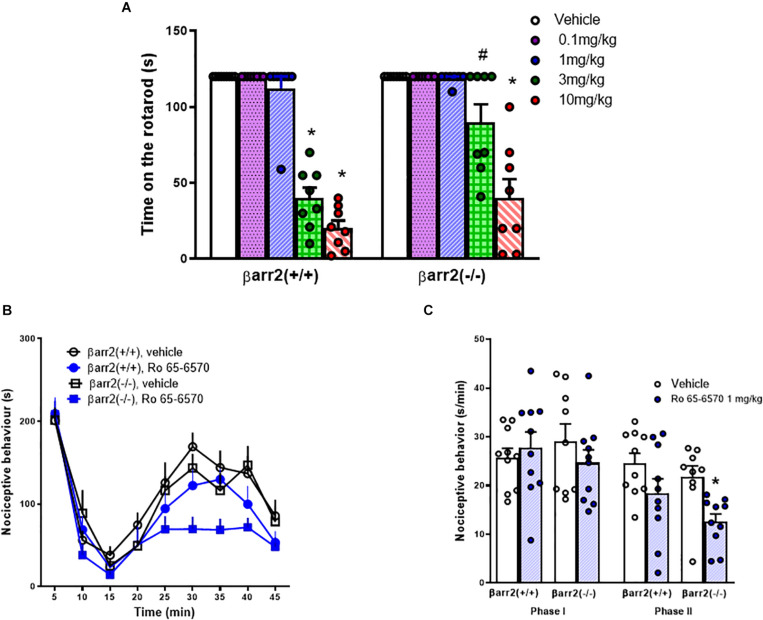
Rotarod and formalin tests in C57BL/6J βarr2(+/+) and βarr2(−/−) mice, effect of Ro 65-6570. **(A)** Time spent on the rotarod, each point represents the mean ± sem of 8 mice/group. Ro 65-6570 0.1–10 mg/kg, injected i.p. Two-way ANOVA treatment x genotype revealed an effect of treatment, genotype, and their interaction [*F*_(4, 70)_ = 78.77, *F*_(1, 70)_ = 13.68, *F*_(4, 70)_ = 5.30]. **(B)** time course of formalin−induced pain behavior. **(C)** cumulative formalin−induced pain behavior during the I° and II° phases. Each point represents the mean ± sem of 10 mice/group. Ro 65-6570 1 mg/kg, injected i.p. Two-way ANOVA (treatment × genotype) revealed an effect of Ro 65-6570 in the second phase *F*_(1, 35)_ = 10.72. ^∗^*p* < 0.05 vs. vehicle, # vs. βarr2(−/−), Tukey’s test.

The rotarod test was used to assess motor impairment induced by Ro 65-6570, injected i.p., in βarr2(+/+) and βarr2(***−***/***−***) C57BL/6J mice. As shown in Figure 3A, no differences were detected between βarr2(+/+) and βarr2(***−***/***−***) mice treated with vehicle on the rotarod. Ro 65-6570 evoked a significant impairment of motor performance of βarr2(+/+) at the doses of 3 and 10 mg/kg. On the other hand, in βarr2(***−***/***−***) mice, Ro 65-6570 significantly reduced the time spent on the rod only at the highest dose.

In the formalin test in C57BL/6J mice, an intraplantar injection of 20 μl of 1.5% formalin solution into the dorsal surface of the right hind paw produced a biphasic nociceptive response: the I° phase started immediately after formalin injection and lasted for 10 min, while the II° phase was prolonged, starting approximately 15–20 min after the injection and lasting for about 40 min. No differences were detected in βarr2(+/+) and βarr2(***−***/***−***) mice treated with vehicle. Ro 65-6570, 1 mg/kg, i.p., did not modify the I° phase of the test in both genotypes. In the II° phase of the assay, Ro 65-6570 significantly reduced nociceptive behavior of βarr2(***−***/***−***) but not βarr2(+/+) mice (Figures 3B,C). Higher doses of Ro 65-6570 were not tested in the formalin assay because of their motor effects on the rotarod.

### Antinociceptive and Locomotor Impairing Effects of MCOPPB, Ro-656570, and AT-403

**FIGURE 4:**
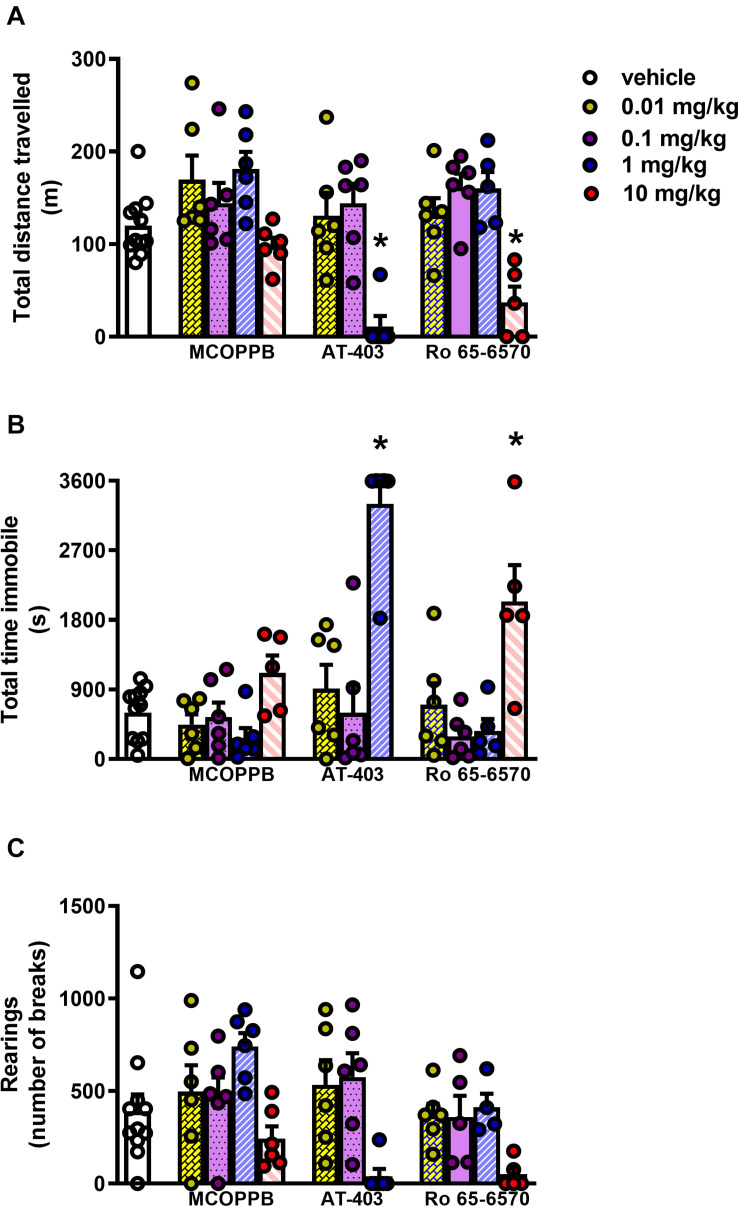
Locomotor activity test in CD-1 mice, dose response curves to AT-403, MCOPPB, and Ro 65-6570 injected i.p. **(A)** cumulative distanced traveled in 60 min, **(B)** total time spent immobile in 60 min, **(C)** cumulative number of rearings performed in 60 min. Each point represents the mean ± sem of 6 mice/group. One-way ANOVA revealed an effect of NOP agonists in the total distance traveled *F*_(11, 63)_ = 8.00, immobility time *F*_(11, 63)_ = 13.53, and number of rearings *F*_(11, 63)_ = 3.76. *p < 0.05 vs. vehicle, Dunnett’s test.

**FIGURE 5:**
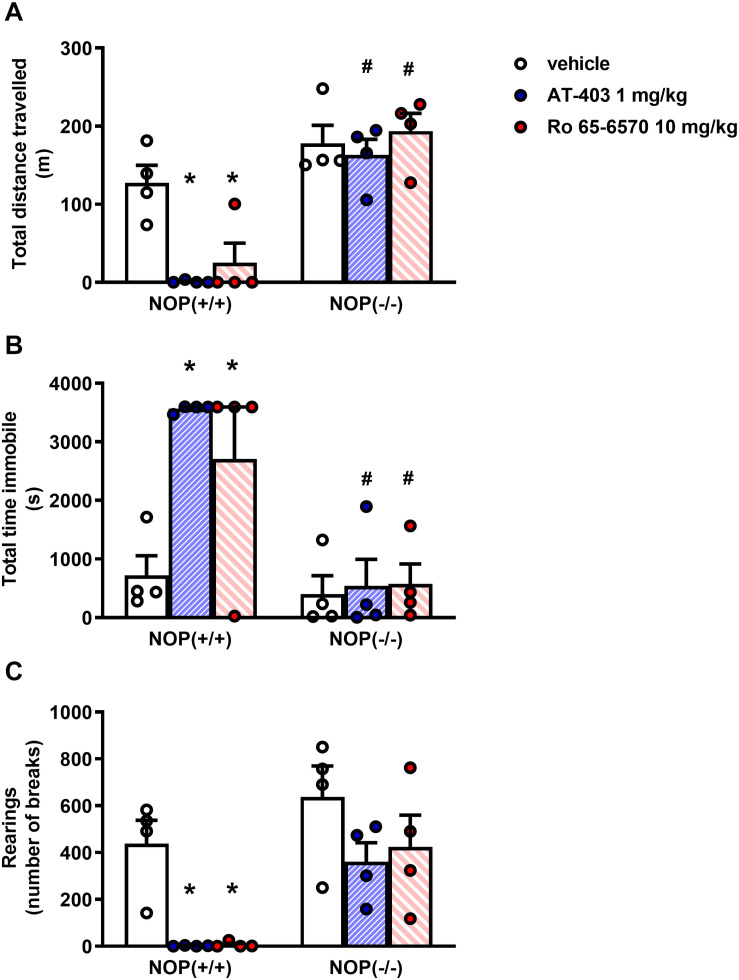
Locomotor activity test in CD-1 NOP(+/+) and NOP(–/–) mice, effects of AT-403 and Ro-656570 injected i.p. **(A)** cumulative distanced traveled in 60 min, **(B)** total time spent immobile in 60 min, **(C)** cumulative number of rearings performed in 60 min. Each point represents the mean ± SEM of 5 mice/group. Two-way ANOVA treatment × genotype revealed an effect of treatment, genotype, and their interaction in the total distance traveled [*F*_(2, 19)_ = 5.83, *F*_(1, 19)_ = 55.83, *F*_(2, 19)_ = 5.08], immobility time [*F*_(2, 19)_ = 5.36, *F*_(1, 19)_ = 22.55, *F*_(2, 19)_ = 4.29], and of number of treatment and genotype in the number of rearings [*F*_(2, 19)_ = 8.75, *F*_(1, 19)_ = 17.99]. *p < 0.05 vs. vehicle, ^#^*p* < 0.05 vs. NOP(+/+), Tukey’s test.

In the locomotor activity test, CD-1 mice treated i.p. with vehicle traveled ∼ 120 m, spent ∼ 600 s immobile, and performed ∼ 500 rearings over the time-course of the experiment. AT-403 and Ro-656570 given i.p. fully inhibited mouse locomotor activity at the dose of 1 and 10 mg/kg, respectively, being inactive at lower doses. On the contrary, MCOPPB given i.p. did not significantly change animal locomotion in the range of doses examined (Figure 4). To investigate the receptor mechanism involved in the locomotor impairing action of AT-403 and Ro 65-6570, the drugs were reassessed in CD-1 NOP(***−***/***−***) mice. As shown in Figure 5, 1 mg/kg AT-403 and 10 mg/kg Ro 65-6570 fully inhibited locomotion in NOP(+/+) mice but were completely inactive in NOP(***−***/***−***) animals.

**FIGURE 6:**
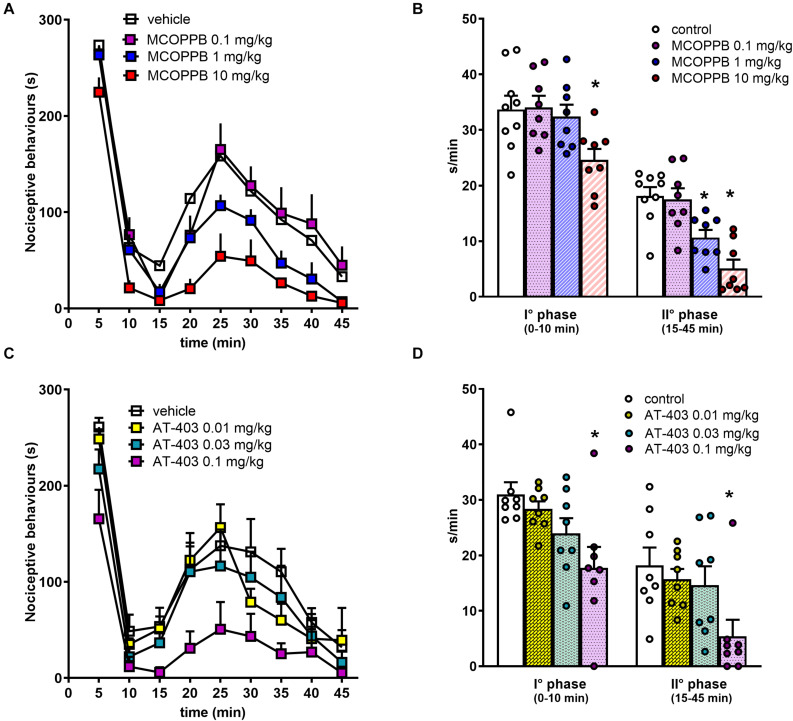
Formalin test in CD-1 mice, dose response curves to MCOPPB and AT-403 injected i.p. **(A,C)** time course of formalin–induced pain behavior. **(B,D)** Cumulative formalin–induced pain behavior during the I° and II° phases. Each point represents the mean ± SEM of 8 mice/group. One-way ANOVA revealed an effect of MCOPPB in the first *F*_(3, 28)_ = 4.05 and second *F*_(3, 28)_ = 13.98 phase, and an effect of AT-403 in the first *F*_(3, 28)_ = 4.71 and second phase *F*_(3, 28)_ = 3.65. *p < 0.05 vs. vehicle, Dunnett’s test.

In CD-1 mice, the intraplantar injection of formalin solution produced effects similar to those described for C57BL/6J mice. MCOPPB produced dose−dependent antinociceptive effects, being active at 1 and 10 mg/kg i.p., for the I° and the II° phase, respectively (Figure 6). AT-403 inhibited both the I° and the II° phase of the assay at 0.1 mg/kg i.p. A complete dose response curve to this compound could not be obtained due to its effects on locomotor performance at higher doses. The dose response curve to Ro 65-6570, injected intravenously (i.v.) in CD-1 mice has been previously performed in our laboratory (Rizzi et al., 2016).

**FIGURE 7:**
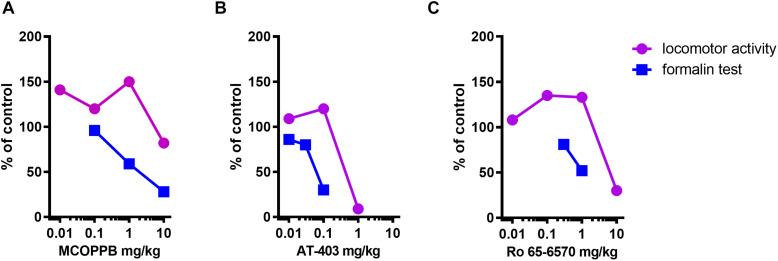
Dose response curves to AT-403 i.p. injected, **(A)**, MCOPPB i.p. injected, **(B)**, and Ro 65-6570 i.p. injected for the LA test and i.v. injected for the formalin assay, **(C)** in the formalin and locomotor activity tests. Data are expressed as% of control, where baseline corresponds to vehicle treated animals. For the LA assay data referred to the total distance traveled, while for the formalin assay data referred to the second phase. These curves (bottom and top constrained to 0 and 100%, respectively) have been used for ED_50_ extrapolation and therapeutic index calculation.

The dose response curves of MCOPPB, AT-403, and Ro 65-6570 in the formalin and locomotor activity assays are shown in Figure 7. To calculate the therapeutic index (antinociceptive vs. locomotor inhibiting effects) of the three NOP agonists, the ED_50_s estimated from these curves were used. Data for Ro-656570, in CD-1 mice, in the formalin assay have been published previously (Rizzi et al., 2016). For the calculation of ED_50_s the bottom and the top of the curves have been constrained to 0 and 100%, respectively. Therapeutic indices of 10, 13, and 9 were calculated for MCOPPB, AT-403, and Ro 65-6570, respectively. No correlation between these values and the bias factors previously reported (Ferrari et al., 2017) were detected (Table 1).

**TABLE 1:** ED_50_, therapeutic index and bias factor for AT-403, MCOPPB, and Ro-656570.

	ED_50_ formalin	ED_50_ locomotor activity	Therapeutic index	Bias factor^*a*^
AT-403	0.07	0.88	13	0.16
MCOPPB	1.10	10.88	10	0.97
Ro-656570	1.04^*b*^	9.79	9	1.64

*ED_50_ in mg/kg. *^*a*^Data from Ferrari et al. (2017). ^*b*^Data from Rizzi et al. (2016).**

## Discussion

This study investigated the possible impact of NOP receptor functional selectivity on antinociceptive vs. motor impairing effects of NOP agonists *in vivo*. βarr2(***−***/***−***) mice displayed slightly increased antinociceptive effects associated with reduced motor impairment in response to the NOP agonist Ro 65-6570. The hypothesis that NOP agonists biased toward G protein might display a larger therapeutic index was tested by comparing the antinociceptive and motor impairing potencies of Ro 65-6570, AT-403, and MCOPPB in wild type mice. Despite 30-fold differences in their bias factor values, NOP agonists displayed a similar therapeutic index (approximately 10) between their antinociceptive vs. hypolocomotor effects.

To investigate the role of βarr2 in the antinociceptive and locomotor inhibiting effects of NOP agonists, experiments were performed with βarr2(***−***/***−***) mice and the NOP selective agonist Ro 65-6570. This compound, identified in Roche laboratories (Wichmann et al., 1999), has been used in several *in vitro* and *in vivo* pharmacological studies (Toll et al., 2016). Importantly the NOP selectivity of Ro 65-6570 *in vivo* has been previously demonstrated both in NOP antagonism and knockout mice studies (Byford et al., 2007; Rutten et al., 2010; Asth et al., 2016; Schiene et al., 2018).

No phenotype differences were detected between βarr2(+/+) and βarr2(***−***/***−***) mice with respect to their sensitivity to formalin and motor performance on the rotarod. This confirms previous studies showing no differences in terms of nociception between βarr2(+/+) and βarr2(***−***/***−***) mice (Bohn et al., 1999; Mittal et al., 2012). Additionally, no differences were reported in the literature between the basal locomotor activity of βarr2(+/+) and βarr2(***−***/***−***) mice (Beaulieu et al., 2005; Urs et al., 2011; Del’Guidice and Beaulieu, 2015; Toth et al., 2018). βarr2(-/-) mice showed different sensitivity to the antinociceptive and motor impairing effects of Ro 65-6570. Specifically, Ro 65-6570 1 mg/kg failed to produce antinociceptive effects in βarr2(+/+) but was effective in βarr2(***−***/***−***) mice. The lack of effect of Ro 65-6570 1 mg/kg in wild-type mice can be ascribed to the fact that C57BL/6J mice are less sensitive to the antinociceptive effects of NOP agonists than CD-1 mice used in our previous studies (Rizzi et al., 2016). Higher doses of Ro 65-6570 could not be tested in the formalin assay because of their motor inhibiting effects. Mice lacking the βarr2 protein were sensitive to the antinociceptive effects of 1 mg/kg Ro 65-6570. This result parallels findings obtained with morphine whose antinociceptive action is potentiated in βarr2(***−***/***−***) mice (Bohn et al., 1999; Azevedo Neto et al., 2020) as well as in phosphorylation-deficient mu knock-in mice (Kliewer et al., 2019). Collectively these results can be interpreted assuming that βarr2 acts [as demonstrated for several GPCRs (Gurevich and Gurevich, 2019)] as a desensitizing element of the antinociceptive response to mu opioid as well as NOP receptor agonists. Of note, NOP receptor phosphorylation and internalization have already been described by different research groups (Spampinato et al., 2001; Corbani et al., 2004; Zhang et al., 2012; Bird et al., 2018; Mann et al., 2019). βarr2(***−***/***−***) mice were slightly less sensitive than βarr2(+/+) mice to the effects of Ro 65-6570 in the rotarod, with the 3 mg/kg dose being active in wild-type animals but not in mice lacking the βarr2 gene. Thus, the βarr2 protein seems to contribute, at least in part, to mediating the inhibitory effect of NOP agonists on locomotion; however, to the best of our knowledge no direct evidence in support of this suggestion is available in the literature. It is worthy of mention that βarr2(***−***/***−***) mice are reported to be less sensitive to the effects of drugs altering locomotion. This has been demonstrated both for stimulants [i.e., amphetamine, apomorphine (Beaulieu et al., 2005), and morphine (Urs et al., 2011)] and for drugs reducing locomotor activity [i.e., lithium (Beaulieu et al., 2008; Del’Guidice and Beaulieu, 2015), lamotrigine and valproate (Del’Guidice and Beaulieu, 2015), and the growth hormone secretagogue receptor 1a antagonist YIL781 (Toth et al., 2018)]. The authors of these studies ascribed this finding to the involvement of βarr2 in the intracellular events that follow dopamine receptor activation.

The experiment with βarr2(***−***/***−***) mice prompted us to hypothesize that NOP agonist biased toward G protein may display a larger therapeutic window in terms of analgesia vs. motor impairment side effects. To test this attractive hypothesis we used three chemically different NOP agonists Ro 65-6570, AT-403, and MCOPPB. DMR experiments performed under the experimental conditions previously described by Malfacini et al. (2018) demonstrated that these compounds act as NOP full agonists with the following rank order of potency MCOPPB > AT-403 > Ro 65-6570. These results are perfectly in line with findings previously obtained in different pharmacological assays including stimulation of [^35^S]GTPγS [binding, calcium mobilization, BRET assay measuring NOP interaction with G protein and βarr2, and the electrically stimulated mouse vas deferens bioassay (Ferrari et al., 2017). Importantly, BRET studies demonstrated that these NOP agonists displayed different G protein vs. βarr2 bias factors; in particular AT-403 (0.16) behaved as an unbiased agonist while MCOPPB (0.97) and Ro 65-6570 (1.64) were biased toward G protein (Ferrari et al., 2017). Similar values of bias factor have been previously reported for the latter compounds (Chang et al., 2015; Malfacini et al., 2015).

In mice MCOPPB and AT-403 produced antinociceptive effects in the formalin assay, being active at 1 and 0.1 mg/kg, respectively. A similar antinociceptive effect was recorded for Ro 65-6570 in the formalin assay at the dose of 1 mg/kg (Rizzi et al., 2016). Of note, the second phase of the assay seems more sensitive to the effect of NOP agonists, suggesting an involvement of the NOP receptor mainly in regulating the inflammatory phase of the test. This hypothesis is corroborated by the fact that NOP antagonists produced pro-nociceptive effects only in the second phase of the formalin test (Rizzi et al., 2006). However, the naturally occurring NOP agonist N/OFQ, after intrathecal administration, was able to reduce both the first and the second phase of the rat formalin assay (Yamamoto et al., 1997, 2000). All the NOP agonists tested produced antinociceptive effects, with the following rank order of potency: AT-403 > MCOPPB = Ro 65-6570. Of note the *in vivo* potency of MCOPPB is relatively low; this is, however, in line with previous studies that investigated its actions on anxiety (Hirao et al., 2008) and mood (Holanda et al., 2019). The low potency of MCOPPB *in vivo* can be possibly ascribed to pharmacokinetic issues. As far as the reduction of locomotor activity is concerned, both AT-403 and Ro 65-6570 significantly reduced mouse locomotion at 1 and 10 mg/kg doses, respectively. This effect is due to the selective activation of the NOP receptor, since it completely disappeared in NOP(***−***/***−***) mice. Differently, MCOPPB failed to produce significant effects on locomotor activity up to 10 mg/kg, in line with previous findings (Hirao et al., 2008). The reason for this lack of effect of MCOPPB on locomotion is not known. We can speculate that, despite the NOP occupancy higher than 50% measured in the whole mouse brain (Hayashi et al., 2009), at 10 mg/kg i.p. this compound is not able to reach, at least in sufficient concentrations, those brain areas important for the hypolocomotor effects of NOP agonists (Marti et al., 2004, 2009; Narayanan et al., 2004; Byford et al., 2007).

What is clear from the present experiments is that NOP agonists displayed the same therapeutic index with hypolocomotor doses being approximately 10-fold higher than antinociceptive doses. In particular, no differences were detected between the unbiased agonist AT-403 and the G protein biased agonist Ro 65-6570, suggesting that the different ability of inducing G protein vs. βarr2 recruitment of a NOP agonist cannot be applied to predict the antinociceptive vs. hypolocomotor properties. These pharmacological findings fail to support the hypothesis that NOP receptor functional selectivity (i.e., G protein vs. βarr2 coupling) can be a useful strategy to obtain better tolerated analgesics, however some caution should be adopted in the interpretation of these results. In fact, the ligands used in this study display bias factor values of 0.94 (MCOPPB) and 1.64 (Ro 65-6570), meaning that the difference in the recruitment of G protein vs. βarr2 is of 10 and 40-fold, respectively. Therefore, it is possible that these values of bias factor are too low to have significant impact *in vivo*. For instance, it has been reported that MCOPPB and the Ro 65-6570 analog Ro 64-6198, are able to induce NOP receptor phosphorylation and internalization similar to the endogenous NOP agonist N/OFQ, both *in vitro* and *in vivo* (Mann et al., 2019), demonstrating that βarr2 dependent biological events can occur after the treatment with these NOP ligands. It is possibly worthy of mention that the NOP agonist cebranopadol displays a very large bias toward G protein *in vitro* and acts *in vivo* as a potent antinociceptive drug devoid of inhibitory actions on mouse motor performance (Rizzi et al., 2016). However, the *in vivo* pharmacological actions of cebranopadol cannot be exclusively attributed to its NOP agonist activity since this molecule is able to simultaneously activate NOP and classical opioid receptors (Calo and Lambert, 2018; Tzschentke et al., 2019). Unfortunately, nothing is known about the functional selectivity of these compounds for the different types of inhibitory G proteins that can be activated by the NOP receptor, this notion could help to explain a possible significance of biased agonism for the NOP receptor.

Finally we would like to underline some limitations of the present study. First, the estimate of the NOP agonist therapeutic indexes is far from being optimal since some ED_50_ values could be only roughly estimated. Second, some comparisons between set of data have to be looked at with caution because different strains (C57BL/6J vs. CD-1) of mice and drug route of administration (i.p. vs. i.v.) have been used. Third, no information is available on the pharmacokinetic properties of the drugs used in this study; such information could significantly contribute to correctly interpret *in vivo* findings. However, at least in our opinion, the above mentioned limitations do not substantially compromise the major finding of the study that is lack of correlation between *in vitro* biased agonism and *in vivo* therapeutic index of NOP agonists.

## Conclusion

In conclusion, this study investigated the possible impact of functional selectivity (G protein vs. βarr2) on the ability of NOP agonists to promote analgesic vs. motor impairing effects. Studies with βarr2(***−***/***−***) mice suggested that bias toward G protein might be associated with a wider therapeutic index, however, this hypothesis was not corroborated by pharmacological studies that demonstrated how unbiased and G protein biased NOP agonists display similar antinociceptive vs. motor impairing properties.

## Data Availability Statement

The raw data supporting the conclusions of this article will be made available by the authors, without undue reservation, to any qualified researcher.

## Ethics Statement

The animal study was reviewed and approved by the Animal Welfare Body of the University of Ferrara and Italian Ministry of Health (License N° 302/2017).

## Author Contributions

CR and GC designed this study. CR, JA, CS, and DM performed the experiments and conducted the statistical analysis. SP and NZ synthesized Ro 65-6570 and AT-403, respectively. CR and GC prepared the manuscript. JA, CR, CS, DM, SP, NZ, and GC revised the manuscript into its finalized form. All authors contributed to the article and approved the submitted version.

## Conflict of Interest

NZ was an employee of Astraea Therapeutics. The remaining authors declare that the research was conducted in the absence of any commercial or financial relationships that could be construed as a potential conflict of interest.
